# Diagnosis of benign and malignant peripheral lung lesions based on a feature model constructed by the random forest algorithm for grayscale and contrast-enhanced ultrasound

**DOI:** 10.3389/fonc.2024.1352028

**Published:** 2024-03-11

**Authors:** Hong Wei, Yichun Wang, Jinyao Li, Yanyan Wang, Longdi Lu, Jiawei Sun, Xiaolei Wang

**Affiliations:** In-Patient Ultrasound Department, The second Affiliated Hospital of Harbin Medical University, Surgeons’ Hall, Harbin, China

**Keywords:** peripheral pulmonary lesions, contrast-enhanced ultrasound, benign, malignant, random forest

## Abstract

**Rationale and objectives:**

To construct a predictive model for benign and malignant peripheral pulmonary lesions (PPLs) using a random forest algorithm based on grayscale ultrasound and ultrasound contrast, and to evaluate its diagnostic value.

**Materials and methods:**

We selected 254 patients with PPLs detected using chest lung computed tomography between October 2021 and July 2023, including 161 malignant and 93 benign lesions. Relevant variables for judging benign and malignant PPLs were screened using logistic regression analysis. A model was constructed using the random forest algorithm, and the test set was verified. Correlations between these relevant variables and the diagnosis of benign and malignant PPLs were evaluated.

**Results:**

Age, lesion shape, size, angle between the lesion border and chest wall, boundary clarity, edge regularity, air bronchogram, vascular signs, enhancement patterns, enhancement intensity, homogeneity of enhancement, number of non-enhancing regions, non-enhancing region type, arrival time (AT) of the lesion, lesion-lung AT difference, AT difference ratio, and time to peak were the relevant variables for judging benign and malignant PPLs. Consequently, a model and receiver operating characteristic curve were constructed with an AUC of 0.92 and an accuracy of 88.2%. The test set results showed that the model had good predictive ability. The index with the highest correlation for judging benign and malignant PPLs was the AT difference ratio. Other important factors were lesion size, patient age, and lesion morphology.

**Conclusion:**

The random forest algorithm model constructed based on clinical data and ultrasound imaging features has clinical application value for predicting benign and malignant PPLs.

## Introduction

Lung cancer conspicuously dominates as the principal male malignancy in terms of incidence and mortality rates, and its trajectory in female cancer incidence is on an unmistakable upward trend (1). Globally, approximately 2.2 million lung cancer cases emerged in 2020, culminating in roughly 1.8 million fatalities (1, 2). Thus, the early detection of malignant lung tumors and timely clinical intervention can effectively reduce mortality and improve patient prognosis. Low-dose computed tomography (CT) has garnered recognition as a potent tool for early lung cancer detection and screening, presenting a statistically significant reduction in lung cancer mortality of approximately 20% when juxtaposed with conventional chest X-ray examinations (3). Nonetheless, the propensity for false positives in CT-based lung cancer diagnosis remains significant (4), with histopathological examination retaining its gold standard status for the diagnosis of malignant lung lesions.

Considering that ultrasound can provide characteristic insights into lesions near the pleura, and the emergence and maturity of contrast-enhanced ultrasound (CEUS) technology, researchers have recently gone beyond simple grayscale ultrasound to deeply study the microvascular supply distribution and dynamic perfusion process of peripheral pulmonary lesions (PPLs) to clarify their vascular characteristics (5–7). Although some studies have used quantitative and qualitative parameters to effectively diagnose benign and malignant PPLs (8–10), few have comprehensively analyzed and evaluated the weight of multiple features of PPLs on grayscale ultrasound and CEUS.

Random forest is a machine learning algorithm that is an ensemble learning model composed of multiple decision trees. Compared with logistic regression analysis, random forest has significant advantages in dealing with feature correlation, feature selection, and handling nonlinear data (11). Random forest models are more appropriate for datasets with many features.

In this study, the clinical data and features of patients with peripheral lung lesions on grayscale ultrasound and CEUS were prospectively collected. A model was established and validated using the random forest algorithm to evaluate the correlation between the relevant variables and the diagnosis of benign and malignant PPLs, thereby revealing the significant factors that distinguish them.

## Materials and methods

### Patients

**Figure 1:**
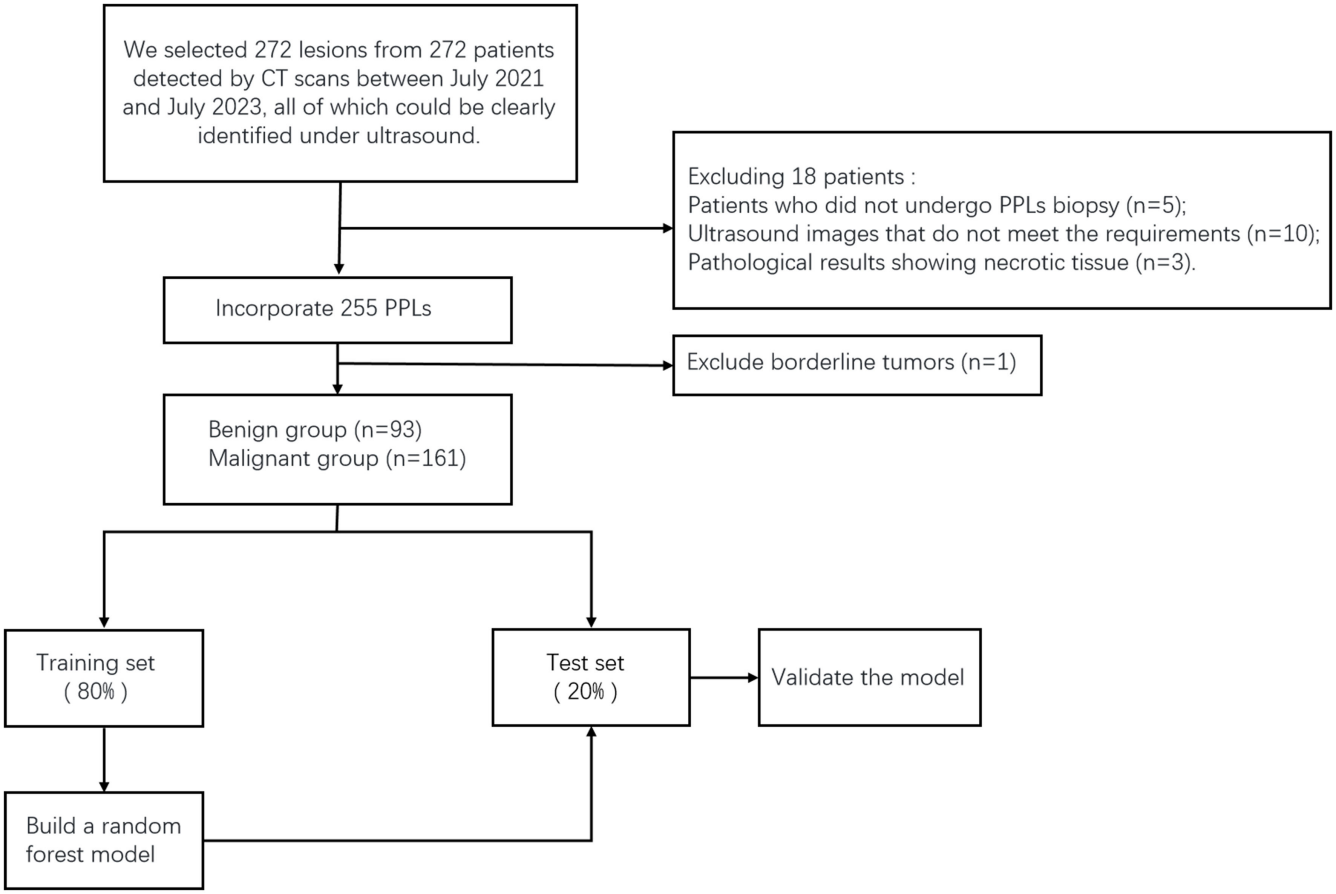
Patient enrollment flowchart.

This study was approved by the Ethics Committee of the Ethics Committee of the Second Affiliated Hospital of Harbin Medical University. We selected 272 lesions from 272 patients detected by CT between October 2021 and July 2023, all of which could be clearly identified using ultrasound. These patients underwent CEUS- and ultrasound-guided percutaneous biopsies in our department. The exclusion criteria were as follows: 1. patients who could not tolerate or cooperate with surgery; 2. severe cardiovascular diseases, such as myocardial infarction, advanced hypertension, and circulatory failure; 3. coagulation disorders or severe bleeding tendencies; and 4. allergies to contrast agents. Before undergoing CEUS and percutaneous biopsy, each patient or a family member signed an informed consent form. Five patients who did not undergo CEUS or biopsy were excluded. Ten patients were excluded because of poor ultrasound image quality and inability to undergo TIC curve analysis. Three patients were excluded because of the lack of pathological results. During the study period, we closely followed up patients with negative biopsy results for 3 to 6 months. If the lesion decreased or disappeared in subsequent CT scans, it was considered benign. If the lesion remained unchanged or increased in size in CT scans, we performed another biopsy to confirm. We obtained the final pathological results of the lesions, with one patient excluded because the pathological results showed a borderline hemangiopericytoma-like fibrohistiocytic tumor. In total, 254 patients (254 lesions) were included in this study (**Figure 1**). The sex, age, and smoking history of the patients were also recorded.

### Image acquisition and analysis

Routine ultrasonography and CEUS were performed using the Aixplorer Color Doppler Ultrasonic Diagnostic Device (SuperSonic Imaging, Aix-en-Provence, France) and the Aplio i900 Color Doppler Ultrasonic Diagnostic Device (Canon Medical, Japan). The devices were equipped with XC6-1 and i8CX1 convex probes. Depending on the location of the lesion, an appropriate position was selected for scanning through the intercostal space. Initial grayscale ultrasonography was performed to observe the morphological characteristics of the lesion such as size (the sum of the maximum diameter parallel to the chest wall and the maximum diameter perpendicular to the chest wall), shape (wedge/circular/fusiform/hemisphere/irregular), angle between the lesion border and chest wall (as long as one angle is obtuse, the parameter is classified as obtuse), boundary clarity (clear or unclear), edge regularity (regular or irregular), and the presence of an air bronchogram sign. The ultrasound contrast agent (UCA) used was SonoVue (Bracco Company). After adjusting the optimal section, the mode was switched to contrast enhancement (mechanical index, <0.1). A rapid injection of 2 mL of UCA was administered into the elbow vein, followed by a flush with 5 mL of saline, during which timing and dynamic image storage began.

**Figure 2:**
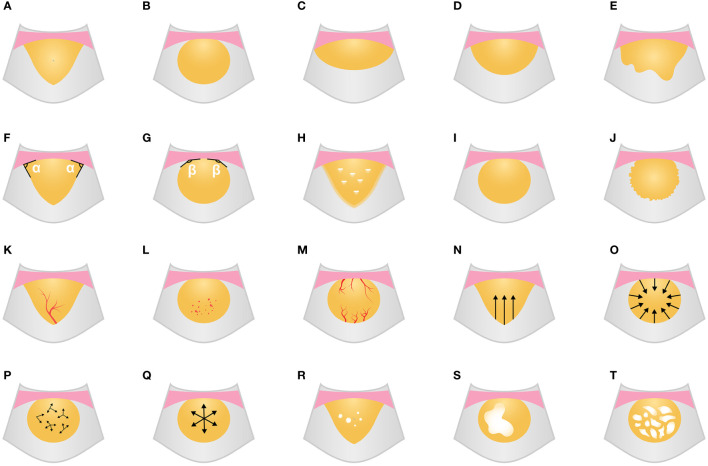
Grayscale ultrasound and CEUS feature schematic diagram. **(A-E)** Shape (**A**, wedge; **B**, circular; **C**, fusiform; **D**, hemisphere; **E**, irregular). **(F, G)** Angle between lesion border and chest wall (**F**, acute; **G**, obtuse). **(H)** Boundary blurred with air bronchogram. **(I, J)** Edge regularity (**I**, regular; **J**, irregular). **(K-M)** Vascular signs (**K**, tree-like; **L**, dot-like; **M**, curl-like). **(N-Q)** Enhancement patterns (**N**, unidirectional emitting; **O**, converging; **P**, diffuse; **Q**, centrifugal). **(R-T)** the number and type of non-enhancing region (**R**, multiple and small piece; **S**, single and large piece; **T**, mesh). CEUS, contrast-enhanced ultrasound.

The enhancement characteristics of the lesion included vascular signs (morphological characteristics of the earliest enhanced blood vessels in the lesion: tree-like/dot-like/curl-like/mixed), enhancement patterns (the way the UCA enters the lesion: unidirectional emitting/converging/centrifugal/diffuse/mixed), and enhancement intensity (the enhanced degree of air-filled lung tissues was defined as hyper-enhancement, and the enhanced degree of thoracic wall muscle was defined as hypo-enhancement: hyper-, iso-, or hypo-enhancement), homogeneity of enhancement (homogeneous or inhomogeneous), number of non-enhancing regions (none/single/multiple), and non-enhancing region type (the morphological characteristics of the non-enhancing region: small piece/large piece/mesh). The definitions of the qualitative parameters for grayscale ultrasound and ultrasound contrast imaging are shown in **Figure 2**.

**Figure 3:**
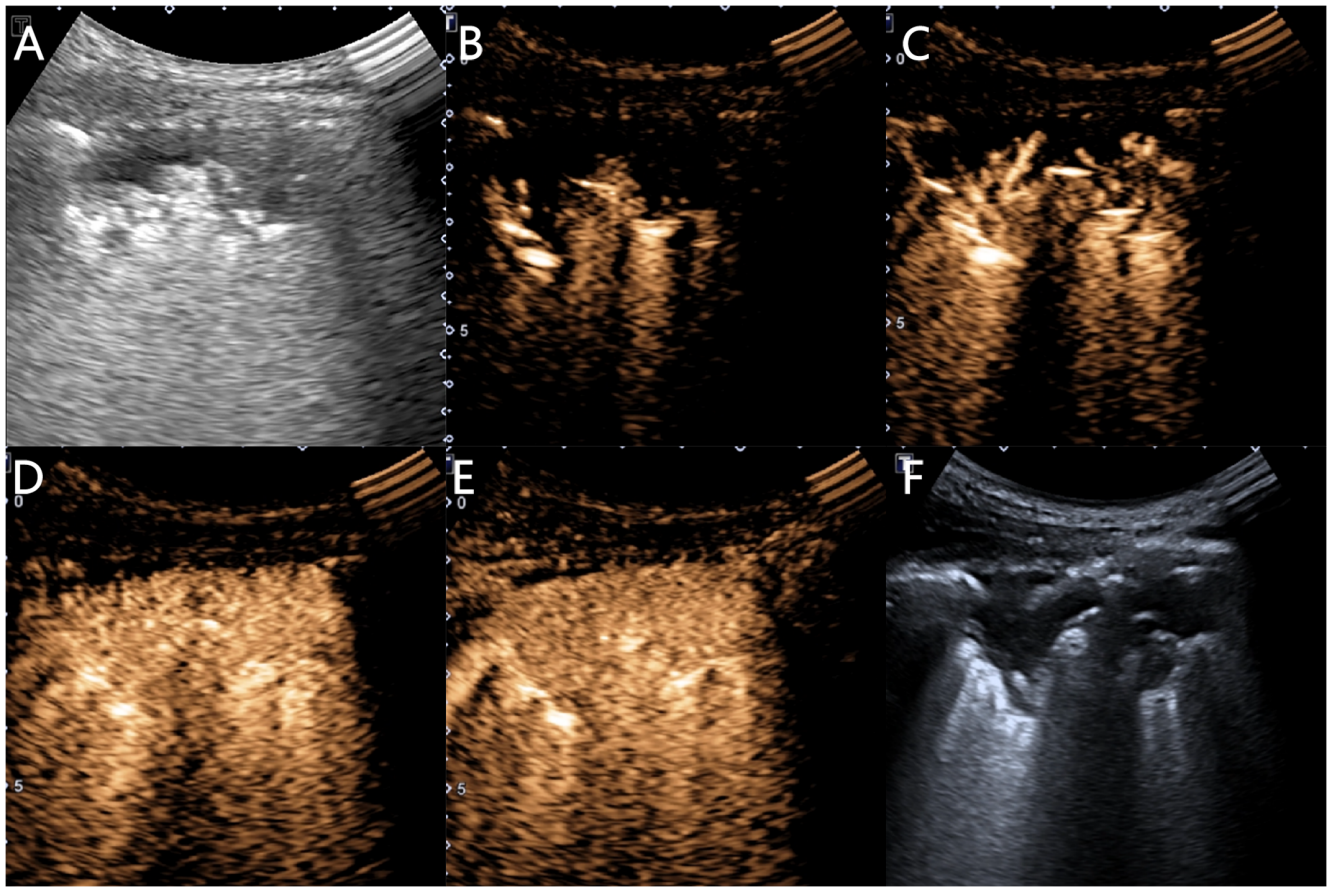
Ultrasound images of an 81-year-old female patient with pneumonia. **(A)** A hypoechoic solid lesion with an irregular shape and blurred edges, measuring 5.4 × 1.9 cm, can be seen in the right lung. **(B)** At 4.1 s, the inflated lung tissue begins to enhance. **(C)** At 5.0 s, the lesion begins to enhance unidirectionally in a branching radicular pattern. **(D)** At 12.6 s, the chest wall begins to enhance. **(E)** The lesion shows uniform isointense enhancement, with no necrosis inside. **(F)** A 16G needle is used for biopsy of the lesion.

**Figure 4:**
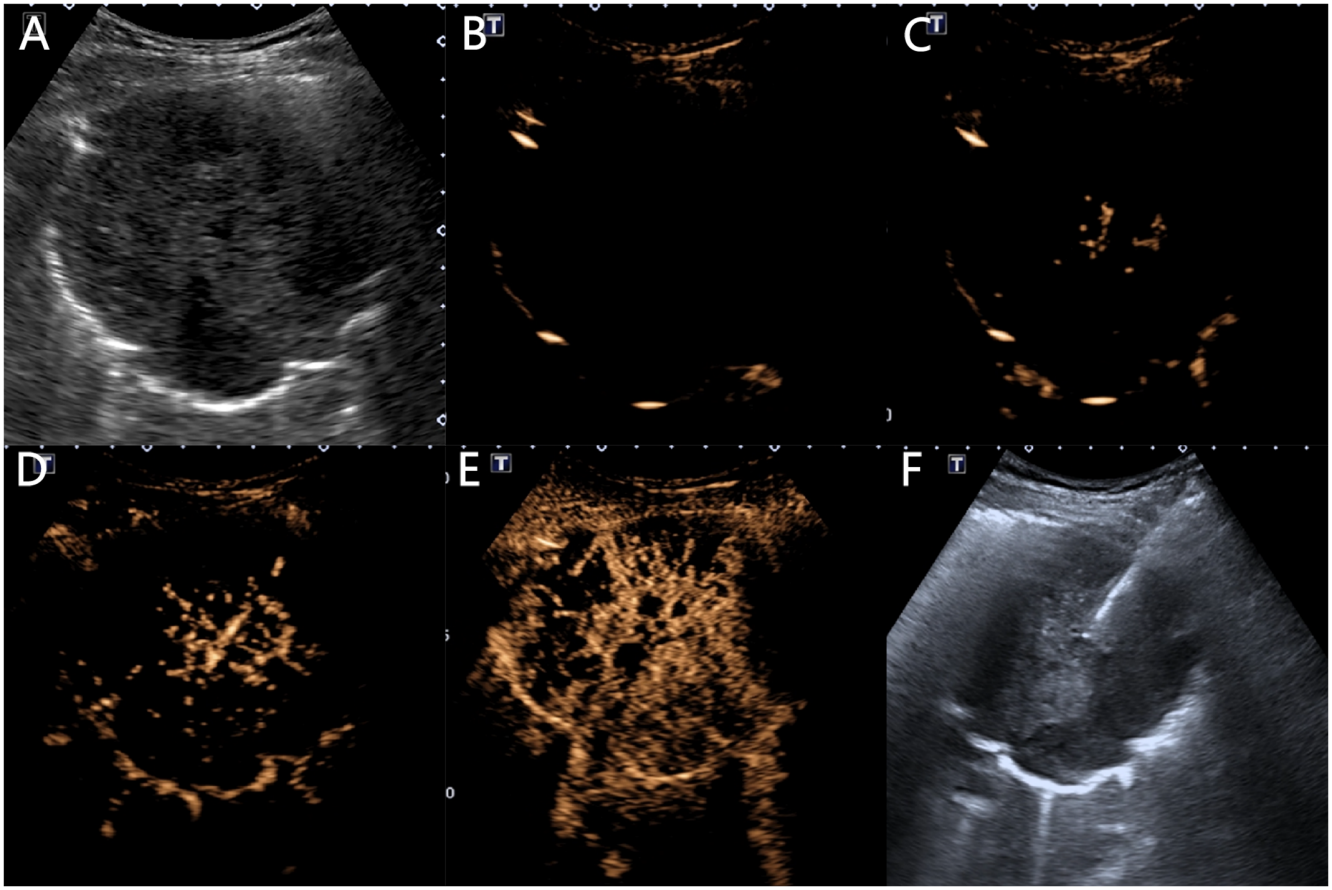
Ultrasound manifestations of a 64-year-old male patient with squamous cell carcinoma. **(A)** A low-echo solid mass of 10.1 × 8.3 cm can be seen in the right lung, with a circular shape and clear boundaries. **(B)** At 2.8 s, the lung tissue begins to enhance with inflation. **(C)** At 7.6 s, the lesion begins to centrifugally enhance in a patchy manner. **(D)** At 8.8 s, the chest wall begins to enhance. **(E)** The lesion shows heterogeneous low enhancement, with multiple necrotic areas visible inside, and the enhanced morphology is sieve-like. **(F)** A 16G puncture needle is used to puncture and sample the central non-necrotic area of the lesion.

Using the ultrasound contrast quantitative analysis software SonoLiver, the contrast film segments were analyzed by placing the regions of interest in the earliest enhanced inflated lung tissue, lesion, and chest wall area, obtaining TIC curves, and finally obtaining the arrival time (AT) of the lung tissue (the time it takes for the contrast agent to enter the normally inflated lung tissue from injection), lesion AT (the time it takes for the contrast agent to reach the lesion), chest wall AT (the time it takes for the contrast agent to reach the chest wall after injection), lesion-lung AT difference (the AT difference between the lesion and the normally inflated lung tissue), AT difference ratio (the ratio of “AT difference between the lesion and the normally inflated lung tissue” to “AT difference between thoracic wall and the normally inflated lung tissue”), time to peak (TTP) (the time it takes for the contrast agent to reach its maximum enhancement at the lesion site from injection), and rising time (RT) (the difference is between the time it takes for the contrast agent to reach its maximum enhancement at the lesion and when the lesion starts to enhance) (**Figures 3**, **4**).

### Reference standards

Under the circumstance where the pathological results for the lesion were unclear, a young physician with five years of diagnostic experience reached a consensus with two chief physicians, each with over ten years of diagnostic experience, on the placement of the area of interest and observation of the imaging characteristics of the lesion.

### Ultrasound-guided percutaneous biopsy

Two physicians with ten years of interventional experience performed lung lesion biopsies. When examining conventional ultrasound and CEUS, physicians accurately determined the optimal location, area, needle entry point, and path for biopsy. The area was disinfected with iodine and sterilized, followed by local anesthesia with 2% lidocaine at the relevant site. Under ultrasound guidance, the physicians avoided the necrotic area shown on CEUS, ensuring that the biopsy needle accurately reached the lesion site. Multiple punctures were made on the lesion, and up to 2-3 tissue samples were collected. All tissue samples were fixed in 4% formalin and sent to the pathology department for analysis.

### Statistical analyses

This study utilized SPSS 27.0 and PyCharm software for statistical analysis. Count data were first subjected to the Shapiro-Wilk test for normality: if the data followed a normal distribution, they were presented as mean ± standard deviation and subjected to an independent samples *t*-test; if the data did not follow a normal distribution, they were presented as the median and interquartile range [M (Q1, Q3)] and subjected to the independent samples Mann-Whitney test. Categorical variables are presented as frequencies or percentages and were tested using the chi-squared test. Statistical significance was set at *P* < 0.05.

Next, the statistical data of the benign and malignant lesions were analyzed using univariate logistic regression, selecting ultrasonic parameters with significant differences between benign and malignant changes as candidate variables.

Finally, the random forest algorithm was used for multivariate analysis to select candidate variables. The random forest analysis consists of three steps: 1. establishing a classification model using training set data and training it; 2. evaluating the performance of the model on the validation set through cross-validation and save the optimal model for easy subsequent data uploading and classification calculations; 3. evaluating the model’s classification accuracy on the test set, and the feature importance was ultimately calculated.

## Results

### Patient general information characteristics

Of the 254 participants in our study, 148 were male and 106 were female, with ages ranging from 16 to 89 years. The final diagnostic results are shown in **Table 1**.

**Table 1:** Final diagnosis results.

	Benign lesions (n)		Malignant lesions (n)	
	Pneumonia	68	Adenocarcinoma	77
	Lung abscess	6	Squamous cell carcinoma	43
	Pulmonary tuberculosis	15	Small cell carcinoma	16
	Granulomatous vasculitis	1	Adenosquamous carcinoma	2
	Fibroma	2	Neuroendocrine carcinomas	2
	Cryptococcosis	1	Metastatic carcinomas	2
			Papillary carcinoma	1
			Spindle cell carcinoma	1
			Sarcomatoid carcinoma	1
			Diffuse large B-cell lymphoma	1
			Non-small cell carcinoma lacking specific pathological findings	15
Total		93		161

When comparing sex distribution (*P*=0.315) and smoking history (*P*=0.904) between the malignant and benign groups, we found no statistically significant differences. However, the average age in the malignant group was notably higher than in the benign group [66.0 (60.00~74.00) versus 62.00 (53.00~68.50), *P <* 0.001] (**Table 2**). Moreover, age as a diagnostic criterion yielded an area under the curve (AUC) of 0.64, pinpointing 62.5 years as the optimal age cutoff. Using this cutoff, the sensitivity was 0.69, and the specificity was 0.54.

**Table 2:** Univariate regression analysis of each parameter between the benign and malignant groups.

Characteristics	Benign group	Malignant group	OR (95%CI)	*P* value
Sex (n, %)				
Male	58 (62.4%)	90 (55.9%)	1.00	
Female	35 (37.6%)	71 (44.1%)	1.31 (0.78-2.20)	0.315
Age, years (M, IQR)	62 (53, 68)	66 (60, 74)	1.04 (1.02-1.07)	
Smoking (n, %)				
No	53 (57.0%)	93 (57.8%)	1.00	
Yes	40 (43.0%)	68 (42.2%)	0.97 (0.58-1.62)	0.904
Size (n, %)	8.45 (6.13, 10.92)	11.04 (8.33, 14.80)	1.15 (1.08-1.22)	<.001*
Shape (n, %)				
Wedge	53 (57.0%)	23 (14.3%)	1.00	
Circular	8 (8.6%)	39 (24.2%)	11.23 (4.55-27.76)	<.001*
Fusiform	8 (8.6%)	34 (21.1%)	9.79 (3.93-24.39)	<.001*
Hemisphere	6 (6.5%)	13 (8.1%)	4.99 (1.69-14.76)	0.004*
Irregular	18 (19.4%)	52 (32.3%)	6.66 (3.22-13.76)	<.001*
Angle between lesion border and chest wall (n, %)				
Obtuse	13 (14.0%)	70 (43.5%)	1.00	
Acute	80 (86.0%)	91 (56.5%)	0.21 (0.11-0.41)	<.001*
Boundary clarity (n, %)				
Unclear	45 (48.4%)	23 (14.3%)	1.00	
Clear	48 (51.6%)	138 (85.7%)	5.62 (3.09-10.25)	<.001*
Edge regularity (n, %)				
Irregular	76 (81.7%)	84 (52.2%)	1.00	
Regular	17 (18.3%)	77 (47.8%)	4.10 (2.23-7.54)	<.001*
Air bronchogram (n, %)				
No	48 (51.6%)	129 (80.1%)	1.00	
Yes	45 (48.4%)	32 (19.9%)	0.26 (0.15-0.46)	<.001*
Vascular sign (n, %)				
Tree-like	22 (23.7%)	22 (13.7%)	1.00	
Dot-like	55 (59.1%)	60 (37.3%)	1.09 (0.54-2.19)	0.806
Curl-like	7 (7.5%)	68 (42.2%)	9.71 (3.66-25.80)	<.001*
Mixed	9 (9.7%)	11 (6.8%)	1.22 (0.42-3.53)	0.711
Enhancement patterns (n, %)				
Unidirectional emitting	29 (31.2%)	32 (19.9%)	1.00	
Converging	32 (34.4%)	82 (50.9%)	2.32 (1.22-4.44)	0.011*
Centrifugal	1 (1.1%)	6 (3.7%)	5.44 (0.62-47.90)	0.127
Diffuse	17 (18.3%)	25 (15.5%)	1.33 (0.60-2.95)	0.479
Mixed	14 (15.1%)	16 (9.9%)	1.04 (0.43-2.49)	0.937
Enhancement intensity (n, %)				
Hyper-enhancement	39 (41.9%)	40 (24.8%)	1.00	
Iso- enhancement	47 (50.5%)	73 (45.3%)	1.51 (0.85-2.69)	0.156
Hypo-enhancement	7 (7.5%)	48 (29.8%)	6.69 (2.70-16.57)	<.001*
Homogeneity of enhancement (n, %)				
Inhomogeneous	32 (34.4%)	125 (77.6%)	1.00	
Homogeneous	61 (65.6%)	36 (22.4%)	0.15 (0.09-0.27)	<.001*
The number of non-enhancing region (n, %)				
None	39 (41.9%)	60 (37.3%)	1.00	
Single	34 (36.6%)	34 (21.1%)	0.65 (0.35-1.21)	0.176
Multiple	20 (21.5%)	67 (41.6%)	2.18 (1.15-4.14)	0.017*
Non-enhancing region type (n, %)				
None	39 (41.9%)	60 (37.3%)	1.00	
Small piece	22 (23.7%)	21 (13.0%)	0.62 (0.30-1.28)	0.195
Large piece	26 (28.0%)	34 (21.1%)	0.85 (0.44-1.63)	0.624
Mesh	6 (6.5%)	46 (28.6%)	4.98 (1.94-12.78)	<.001*
Lung AT (M, IQR)	4.40 (2.80, 7.05)	4.20 (3.15, 5.75)	0.96 (0.87-1.06)	0.388
Lesion AT (M, IQR)	6.40 (4.35, 9.95)	8.40 (6.70, 10.30)	1.16 (1.07-1.27)	<.001*
Chest AT (M, IQR)	10.80 (9.00, 14.20)	11.20 (9.60, 4.15)	1.01 (0.95-1.08)	0.732
AT difference (M, IQR)	1.60 (1.10, 2.90)	3.80 (2.45, 5.10)	1.83 (1.51-2.21)	<.001*
AT difference ratio (M, IQR)	0.28 (0.17, 0.47)	0.62 (0.46, 0.76)	81.12 (22.87-287.67)	<.001*
TTP (M, IQR)	13.14 ± 4.81	14.73 ± 4.12	1.09 (1.02-1.16)	0.007*
RT (M, IQR)	5.60 (4.05, 7.95)	5.90 (4.40, 7.45)	1.02 (0.92-1.13)	0.770

M, median; IQR, interquartile range; AT, arrival time; TTP, time to peak; RT, rising time.

*P < 0.05.

### Benign and malignant group gray-scale ultrasonic features

Univariate logistic regression analysis showed significant differences between the benign and malignant groups in lesion size, shape, angle between the lesion border and chest wall, boundary clarity, edge regularity, and air bronchogram sign (*P* < 0.05). The median lesion size in the malignant group was 11.04 cm, and the median lesion size in the benign group was 8.45 cm. The AUC of lesion size was 0.68, the optimal cut-off value was 10.19 cm, sensitivity was 0.58, and specificity was 0.71. Malignant lesions were mostly irregular, circular, and fusiform, whereas benign lesions were mostly wedge-shaped. Benign lesions were mostly at acute angles with the chest wall, whereas malignant lesions were equally frequent at acute and obtuse angles with the chest wall. The boundaries of malignant lesions were mostly clear, whereas the frequencies of clear and unclear boundaries of benign lesions were similar. The boundaries of benign lesions were mostly irregular, whereas the regular and irregular frequencies of the boundaries of malignant lesions were similar. Malignant lesions mostly did not have air bronchograms, whereas the frequencies of benign lesions with or without air bronchograms were similar (**Table 2**).

### Benign and malignant group ultrasound contrast-enhanced features

In the CEUS imaging of benign lesions, imaging is mostly mottled and arborescent, while malignant lesions are mostly curled and mottled (*P <* 0.05). Malignant lesions often showed convergent enhancement, whereas benign lesions often showed unidirectional radiative enhancement and convergent enhancement (*P* < 0.05). Benign lesions usually have iso-enhancement or hyper-enhancement effects, whereas malignant lesions usually have iso-enhancement effects. However, the proportion of malignant lesions was significantly higher in the hypoenhancement group (*P* < 0.05). Malignant lesions often exhibit inhomogeneous enhancement, whereas benign lesions often exhibit homogeneous enhancement. Benign lesions usually had a single or no non-enhancing area, whereas malignant lesions usually had multiple non-enhancing areas (*P* < 0.05). In addition, malignant lesions typically had a reticular distribution in non-enhancing areas (*P* < 0.05). Significant statistical differences in the quantitative features on enhanced ultrasound were observed between the benign and malignant lesion groups, such as lesion AT, lesion-lung AT difference, AT difference ratio, and TTP (*P*<0.05) (**Table 2**).

The AUC of lesion AT was 0.64, and the optimal cutoff value was 6.05 s. Meanwhile, the sensitivity was 0.81, and the specificity was 0.48. The AUC of lesion-lung AT was 0.78, and the optimal cutoff value was 2.45 s. Meanwhile, the sensitivity was 0.75, and the specificity was 0.71. The AUC of the AT difference ratio was 0.77, and the optimal cut-off value was 0.45. Meanwhile, the sensitivity was 0.75, and specificity was 0.75. The AUC of TTP was 0.60, and the optimal cutoff value was 12.55 s, while the sensitivity and specificity were 0.70, and 0.53, respectively.

### Random forest modeling results

**Figure 5:**
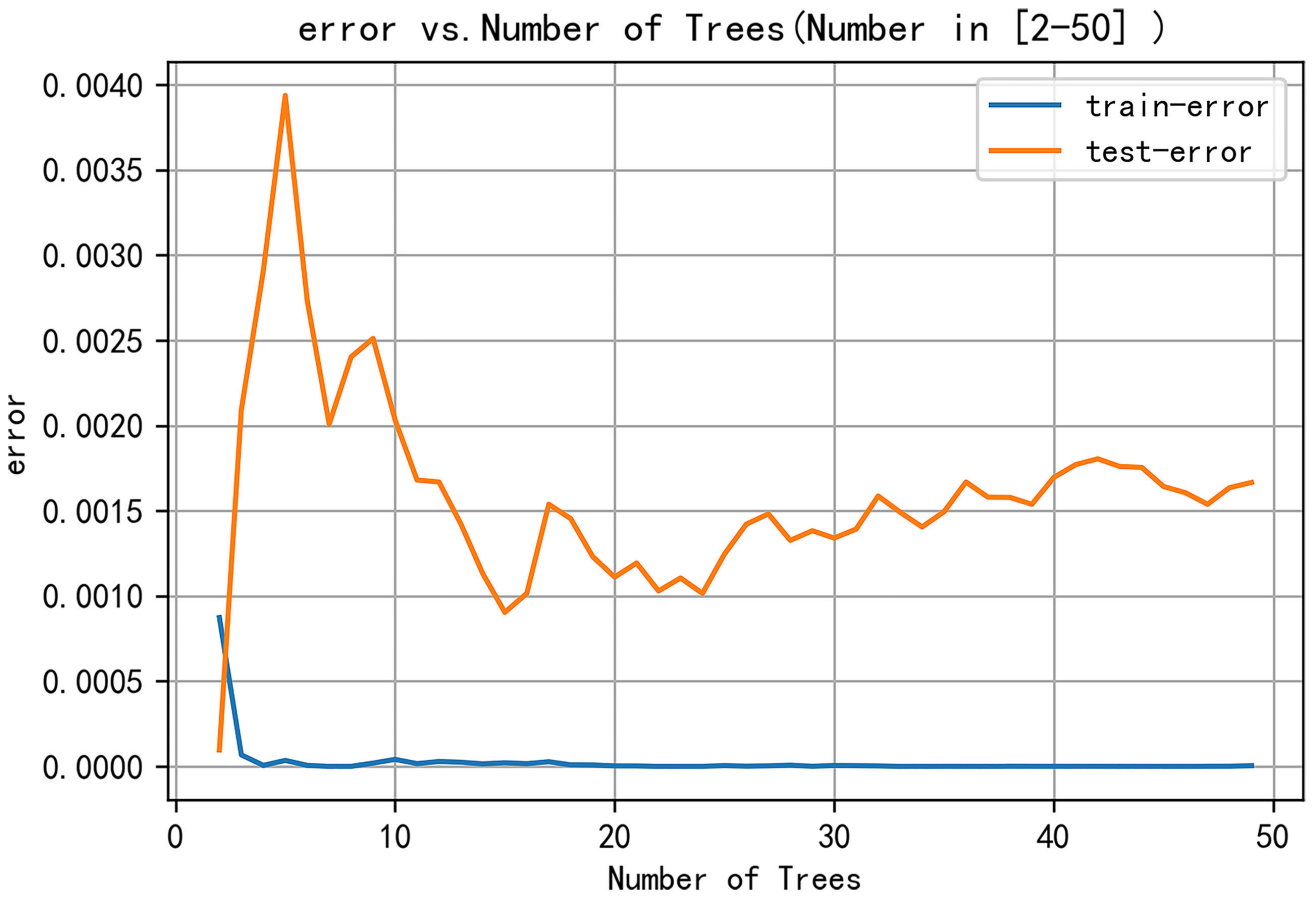
Relationship between model error and number of decision trees.

**Figure 6:**
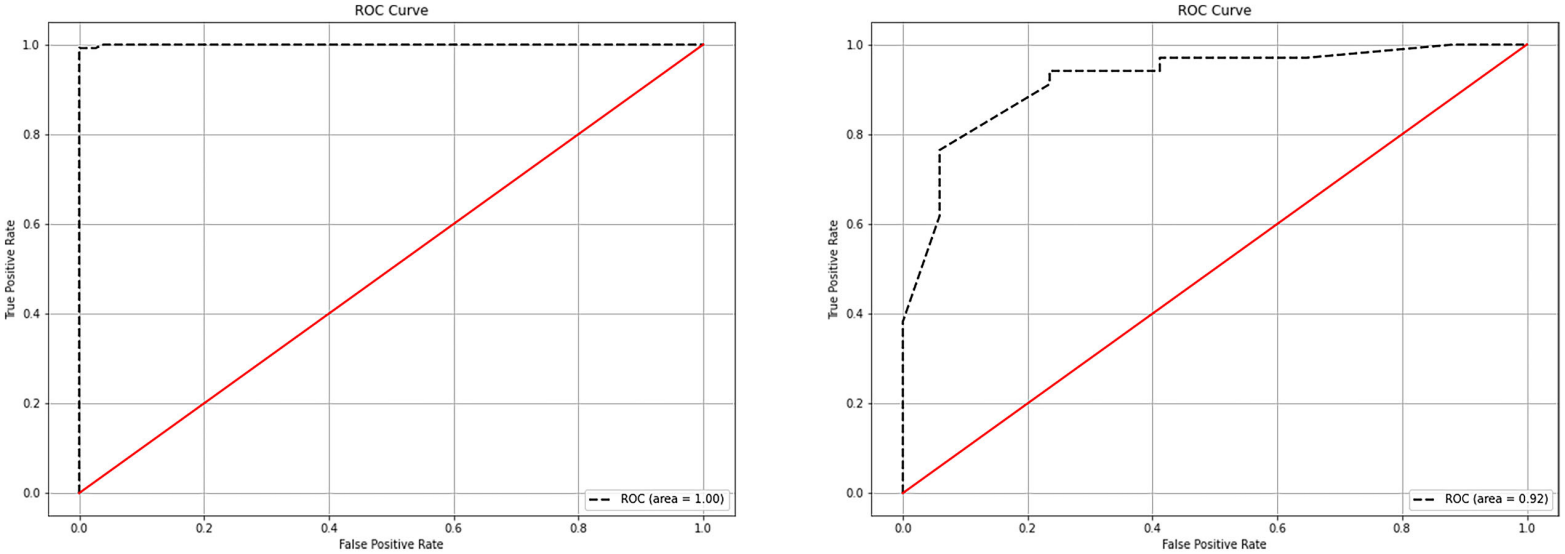
ROC curves of the random forest model in the training and testing sets. **(A)** ROC curves of the random forest model in the training set. **(B)** ROC curves of the random forest model in the testing set. ROC, receiver operating characteristic.

**Figure 7:**
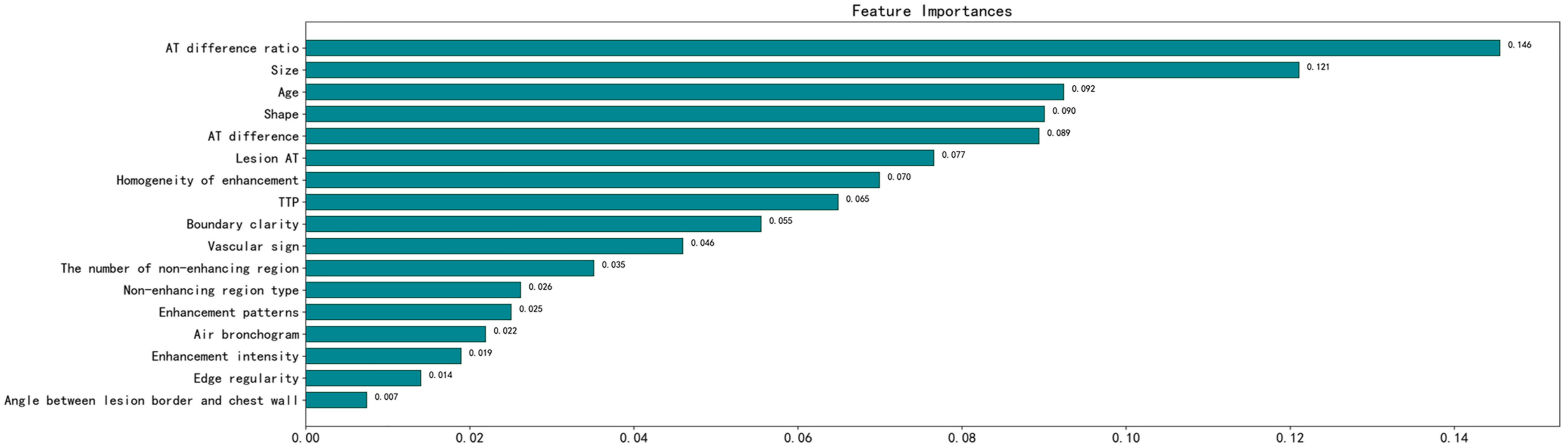
Importance score of predictive variables in the random forest model. AT, arrival time; TTP, time to peak.

The following parameters were set for the random forest model: the random seed number was 1000, and 49 total models were constructed. The relationship between the error and number of trees was then plotted, and the error in the test set was minimized when 15 trees were present (**Figure 5**). Therefore, the random forest model was selected. The area under the receiver operating characteristic (ROC) curve of this model in the training set was 1 and the prediction consistency rate was 0.985. In the test set, the area under the ROC curve was 0.921 and the accuracy rate was 0.882 (**Figure 6**). According to the feature importance coefficient, the importance score of each variable was measured, and the most important factor affecting the discrimination between benign and malignant PPLs was determined to be the AT difference ratio, followed by size, age, and shape (**Figure 7**).

## Discussion

In comparison to previous research methods, we have collected a relatively large amount of general clinical data and ultrasound manifestations of 22 features of PPLs, and extracted 17 features with discriminatory significance between benign and malignant PPLs using logistic regression (9). This increases the likelihood of selecting the optimal features. In comparison to establishing a common multivariate logistic regression model, the advantages of random forests are less susceptible to the influence of multicollinearity between features and can reduce the model’s dependence on a specific training set. Most importantly, random forests are highly useful for situations with a large number of features, helping us to identify the most important features (11). Based on these features, we can comprehensively judge the nature of the lesion, providing a reference for differential diagnosis. We constructed a random forest model in this study based on these 17 indicators to predict the nature of PPLs, with an AUC of 0.92 and an accuracy rate of 88.2% in the test set.

Owing to the dual blood supply characteristics of the lungs, the contrast agent sequentially passes through the right heart, pulmonary circulation, left heart, and systemic circulation after injection into the elbow vein. Consequently, pulmonary artery enhancement occurs earlier than bronchial artery enhancement. Therefore, judging the blood supply source of the lesion by the beginning of the enhancement time can differentiate benign PPLs from malignancies (12). The 2017 ultrasound contrast imaging guidelines released by the European Society of Ultrasound in Medicine and Biology suggest that an initial enhancement time of less than 10 s indicates that the lesion is mainly supplied by the pulmonary artery, which is more common in patients with pneumonia. An enhancement time greater than 7.5 s indicates the possible presence of lung cancer (13). Although benign and malignant PPLs can exhibit different initial enhancement times, an overlap in time between the two may exist, making it impossible to determine the benign or malignant nature of PPLs. In our study, when the lesion AT was greater than 6.05 s, the likelihood of malignant lesions increased. However, due to the different physiological or pathological conditions of patients (such as examination location, chronic heart failure, hyperthyroidism or hypothyroidism, and chronic lung diseases), inconsistent injection speeds of the contrast agent by operators, or even uncoordinated timing by operators, the beginning enhancement times of pulmonary artery and bronchial artery enhancement in patients can be variable (14). Therefore, relying solely on lesion AT for judgment carries risks; thus, the importance ranking in the random forest variables is not high. Therefore, we introduced the difference in the time of arrival of the lesion. When the lesion-lung AT difference was greater than 2.45 s, malignant lesions can be diagnosed. However, in some patients, the difference between the lesion and lung AT values was lower than the critical value. In contrast, the difference between the lesion and chest wall AT values was very close. In our study, such lesions were classified as benign; however, their actual blood supply was closer to that of the chest wall. In this study, we found that the variable importance ranking in the random forest was the first AT difference ratio, which is consistent with the findings of Bi et al. (10, 15). The AT difference ratio was more effective in reducing individual differences than the lesion-lung AT difference. When the AT difference ratio was > 0.45, the likelihood of malignant tumors increased. In addition, statistically significant differences were observed in TTP between the benign and malignant groups, whereas no statistically significant differences existed in RT between the malignant and benign groups, which is consistent with the studies by Bi et al. (15) and Li et al. (9). However, the TTP may also be affected by the lesion AT.

This study was limited to distinguishing between benign and malignant lesions only through quantitative indicators, and we also need to analyze the enhancement patterns of the lesions. In benign lesions, the vascular structure is usually tree-like, the perfusion is unidirectional and radiating, and it shows uniform and significant enhancement. In malignant lesions, the vascular appearance is mostly dotted and curly, and it shows uneven and low enhancement, which is similar to the findings of Li et al. (9) and Shen et al. (16). In benign lesions, the supplying pulmonary artery usually maintains its normal structure; therefore, it presents a tree-like structure radiating from the hilum to the pleural direction on CEUS. Due to inflammatory reactions, vascular dilation, and acceleration of blood flow, the local blood flow increases, resulting in the lesion mostly showing high enhancement. Malignant tumor cells destroy the normal structure of the lung tissue, and the small bronchial arteries gradually replace the hypoxic pulmonary arteries as the tumor grows. The structure of the disordered new vessels produces a large number of anastomotic branches and collaterals, and the distribution is tortuous and uneven, resulting in the enhanced vessels in the lesion showing a dotted or curly appearance and low enhancement (17). However, some studies have shown that the degree of enhancement of malignant lesions is related to pathological results. According to the findings of Findeisen et al. (18) and Wang et al. (19), compared with squamous cell carcinoma, adenocarcinoma has a higher expression of vascular endothelial growth factor, resulting in a higher microvascular density in adenocarcinoma and a higher degree of enhancement. In our study, adenocarcinoma accounted for a higher proportion of tumors in the malignant group, which may have led to malignant lesions showing isoenhancement rather than low enhancement. In addition, we found that the convergent perfusion pattern was more common in malignant lesions, which is consistent with the research of Bi et al. (15) and Fu et al. (20), whereas a local-to-global perfusion pattern was more common in the study by Shen et al. (16). However, these perfusion patterns are related to tumor damage to the blood vessels and the formation of new blood vessels. Moreover, the number and morphology of necrotic areas were significantly different between the benign and malignant groups. However, these qualitative imaging indicators were not highly ranked in the random forest models used in the present study.

The size and shape of the lesion ranked second and fourth, respectively, in terms of the variable importance of the random forests. Unlike previous studies, our clinical experience led to a more detailed description of the shape, which is similar to the results reported by Li et al. In the present study, wedge-shaped lesions were more common among benign lesions, whereas round, spindle, and irregular shapes were more common among malignant lesions. Pneumonia can cause inflammatory cell infiltration and tissue necrosis in local tissues, resulting in vascular occlusion and ischemia of lung tissue in the affected area, thus forming wedge-shaped lesions with blurred boundaries. Malignant cells grow rapidly and spread through blood vessels and bronchi, resulting in increased longitudinal diameter. During growth, surrounding tissues can exert a certain tension, resulting in larger malignant tumors in a ball, spindle, or irregular shape, with clear lesion boundaries (20, 21). The research by Gould et al. (22) shows that the size of the lesion is a key factor, and that the larger the diameter of the lesion, the higher the possibility of malignant lesions. However, this is inconsistent with the studies of Bai et al. (23) and Bi et al. (10), which may be related to small sample datasets or different indicators used to describe the size of the lesion. In this study, we used the sum of the horizontal and vertical diameters to reflect lesion size, considering the size of the lesion in two directions, which can more accurately assess the overall size of the lesion compared with only describing the size of the lesion using the horizontal, vertical, or maximum diameters.

In our study, age ranked third in terms of variable importance in the random forest model, which is in line with the increasing trend in cancer incidence with age. At the same time, smoking is an important risk factor for cancer (1), but no significant correlation was found between smoking and benign and malignant tumors in this study. This may be due to the small sample size or the different proportions of pathological results in the collected cases. According to the study by Loeb et al. (24), smoking is closely related to lung cancer (especially squamous cell carcinoma), but not adenocarcinoma. Importantly, adenocarcinoma accounted for approximately half of the malignant tumors in the present study, which could explain the lack of a significant difference between smoking and benign and malignant tumors.

The limitations of this study are as follows: (1) the number of cases included in this study was small, and the ability of grayscale ultrasound and CEUS to differentiate benign and malignant PPLs still needs further validation with multicenter trials with large sample sizes; (2) the placement of the CEUS region of interest is subjective; (3) this study used ultrasound-guided biopsy, which may lead to sampling errors or insufficiencies in the lesion; and (4) this study did not further analyze the lesion subtypes.

## Conclusions

The random forest algorithm model, based on clinical data and ultrasound imaging features, has clinical application value in predicting benign and malignant PPLs. Combining indicators such as AT difference ratio, size, age, and shape of the lesion can provide a diagnostic basis for differentiating benign and malignant PPLs.

## Data availability statement

The raw data supporting the conclusions of this article will be made available by the authors, without undue reservation.

## Ethics statement

The studies involving humans were approved by Medical Ethics Committee of the Second Affiliated Hospital of Harbin Medical University. The studies were conducted in accordance with the local legislation and institutional requirements. Written informed consent for participation in this study was provided by the participants’ legal guardians/next of kin. Written informed consent was obtained from the individual(s), and minor(s)’ legal guardian/next of kin, for the publication of any potentially identifiable images or data included in this article.

## Author contributions

HW: Data curation, Methodology, Writing – original draft. YCW: Data curation, Writing – original draft, Conceptualization, Writing – review & editing. JL: Data curation, Writing – review & editing, Formal analysis, Investigation. YYW: Data curation, Investigation, Writing – review & editing. LL: Data curation, Formal analysis, Writing – review & editing. JS: Writing – review & editing. XW: Writing – review & editing.
